# Editorial: Advances in green synthesis for drug discovery

**DOI:** 10.3389/fchem.2023.1166887

**Published:** 2023-03-02

**Authors:** Florent Allais, Simone Brogi, Guillermo Raul Castro, Veroniki P. Vidali

**Affiliations:** ^1^ URD Agro-Biotechnologies Industrielles (ABI), CEBB, AgroParisTech, Pomacle, France; ^2^ Department of Pharmacy, University of Pisa, Pisa, Italy; ^3^ Nanomedicine Research Unit (Nanomed), Center for Natural and Human Sciences, Federal University of ABC (UFABC), Santo André, Brazil; ^4^ Max Planck Laboratory for Structural Biology, Chemistry and Molecular Biophysics of Rosario (MPLbioR UNR-MPIbpC), Partner Laboratory of the Max Planck Institute for Biophysical Chemistry (MPIbpC MPG), Universidad Nacional de Rosario—CONICET, Rosario, Argentina; ^5^ Institute of Nanoscience and Nanotechnology, National Centre for Scientific Research “Demokritos”, Athens, Greece

**Keywords:** green chemistry, drug discovery, synthetic approaches, green solvents, green reagents, enzymes for the synthesis of bioactive compounds

In July 2021 we announced a Frontiers Research Topic, hosted by Frontiers in Chemistry, entitled *Advances in green synthesis for drug discovery*, as a part of the Green and Sustainable Chemistry section of the journal. The topic related to the green synthesis methods aimed to highlight novel environmentally safe approaches for synthesizing novel chemical entities with importance in pharmaceutical and pharmacological fields. Due to the growing population and damage from industrial pharmaceutical manufacture, it is well proved that the development of new and improved pharmaceuticals comes with a substantial environmental cost. As a result, one of the most popular study fields at every stage of the drug discovery process is the creation of environmentally friendly technologies. Even while there is opposition to changing the established procedures, the financial benefits that eco-friendly practices provide might spur development in this direction. This means that research in this field still has a lot to offer as the demand for ecologically friendly approaches rises. Green chemistry is a useful instrument that is becoming more significant since drug development raises global living standards. Therefore, this Research Topic focuses on all chemical aspects of the development of bioactive molecules based on green chemistry strategies and represents a practical manual for scientists that work on this area. Due to the factors mentioned above, this Research Topic caught the interest of researchers, receiving a significant number of submissions, with nine original research papers and two reviews published.

Considering the original research articles, Lai et al. reported a convenient method for the synthesis of *N*-heteroaryl esters using *N*-heteroaryl methanols and acyl cyanides through the cleavage of the C–C bond, excluding the use of any transition metal. In fact, the usage of Na_2_CO_3_/15-crown-5 couple allowed preparing different *N*-heteroaryl esters, with great efficiency. Notably, *N*-heteroaryl esters, such as furans, pyridines, pyrazines, quinolines, and thiophenes, constitute vital structures in pharmaceuticals, dyes, flavors, or natural products. The protocol, described in this work, is operationally straightforward and environmentally friendly, only with the production of cyanides as byproducts.

In a fascinating paper, Sayahi et al. exploited the Pd@Py2PZ@MSN as a new and effective catalyst to form C-C bond. In particular, in the described procedure, the authors immobilized palladium onto dipyrido (3,2-*a*:2′,3′-c) phenazine (Py2PZ)-modified mesoporous silica nanoparticles (MSNs), led to the generation of a novel catalyst. A simple procedure was used to create the Py2PZ ligand from the reaction of the raw materials 1,10-phenanthroline-5,6-dione and 3,4-diaminobenzoic acid. The resulting compound was employed for functionalizing MSNs, modifying their surface chemistry so that the palladium could be immobilized. Accordingly, the palladium-immobilized Py2PZ–modified MSNs (Pd@Py_2_PZ@MSNs) were synthesized and characterized using different methods, and their activity and efficiency were studied in C–C bond formation reactions. Particularly, the results were favorable in aqueous medium, and significant yields of desired compounds were achieved. After ten consecutive runs, the catalyst also displayed outstanding reusability and no discernible loss of activity.

Bai et al. used a biosynthetic method for generating novel antimalarial metabolites into the erythrocytes by hydroxylation of the well-known drug dihydroartemisinin (DHA) performed by the organism *Cunninghamella elegans* CICC-40250. The analysis of the resultant compounds and comparison with DHA metabolites in erythrocytes were conducted. The authors using UPLC-MS detected nine DHA derivatives (**M1-M9**) obtained by means of microbial transformation, and some of them such as 7-hydroxydihydroartemisinin (**M1**), 1-deoxydihydroartemisinin (**M8**), and 1-deoxyartemisinin (**M9**) were extracted. These substances were also present in erythrocytes. In order to describe and characterize the substance **M1**, the authors employed X-ray single-crystal diffraction. Furthermore, **M1** showed an interesting *in vitro* antimalarial activity against the *Plasmodium* strain *Pf*3D7 (IC_50_ = 133 nM). Remarkably, the presented work represents the first effort to isolate and characterize the DHA metabolites in erythrocytes through microbial transformation. Accordingly, using environmentally friendly microbial transformation, it is now possible to produce antimalarial drugs as a result of the discovery and synthesis of these compounds.

In another interesting research article, Villano et al. described a simple and adaptable methodology for synthesizing the gut microbiota-derived bioactive metabolite, commendamide (*N*-(3-hydroxypalmitoyl)-glycine) and its derivatives. This substance shares structural similarities with long-chain *N*-acyl-amino acids from the endocannabinoidome, a complicated lipid signaling system that plays significant functions in mammals by, among other things, activating G-protein-coupled receptors (GPCRs) (GPCR G2A/132). Easily available reagents, simple workups, non-halogenated solvents, and small amounts of organic solvents were used to synthesize commendamide in an environmentally friendly and economically feasible manner. Notably, the usage of column chromatography for purifying synthetic intermediate derivatives was moreover frequently avoided due to the high yields seen in several reaction stages. The same synthetic route was also effectively employed for synthesizing deuterated commendamide and an additional secondary commendamide-like metabolite, thus demonstrating the usefulness of the novel synthetic strategy described in the mentioned paper.

Yang et al. described a synthetic protocol to efficiently produce *O*-desmethylvenlafaxine (ODV), the main active metabolite of venlafaxine, a serotonin-norepinephrine reuptake inhibitor (SNRI), which has been marketed for treating mild/severe depression, anxiety, and many different mental diseases, and *O*-desmethylvenlafaxine succinate monohydrate (DVS) in a significant yield and high purity. The five-step synthetic approach was developed using, as the starting material, *p*-hydroxybenzene acetonitrile, followed by condensation of cyclohexanone, deprotection of the phenolic hydroxyl group, cyano reduction, dimethylation, and production of succinic acid salts. The route involved high-yielding steps with low genotoxic impurities (GIs), the use of cheap reagents and solvents, and cost-effective purification procedures. ODV was prepared with 99.92% purity, and DVS was obtained in 71.09% overall yield. According to XRD powder diffraction, the crystalline form of DVS exhibited distinctive peaks at 5, 10, 21, and 26 min. Remarkably, when compared to traditional synthesis methods, the suggested strategy demonstrated an innovative, environmentally friendly method that ensured a great total yield with minimal environmental impact and remarkable residual standards for GIs, improving the safety of the drug.

According to Wang et al., copper fluoride facilitates a simple Hiyama cross-coupling reaction between arylsilanes and thiuram derivatives (tetraalkylthiuram disulfides (TATD) or tetraalkylthiuram monosulfide (TMTM)). This synthetic procedure is a useful option to generate *S*-aryl dithiocarbamates in good yields, showing limited toxic and easily accessible substrates, low-cost promoter, and straightforward performance. The method on the cross-coupling of arylsilanes with TATD or TMTM enhanced by copper allowed for the production of the significant *S*-aryl dithiocarbamates in yields ranging from moderate to good. This simple method allowed for practical and friendly reaction conditions, greatly enhancing functional group compatibility, increasing the range of substrates, and stressing the synthetic use of complicated compounds. The suggested method can provide a strategy to expeditely synthesize *S*-aryl dithiocarbamates from affordable and stable substrates as well as a fresh illustration of how to use Hiyama cross-coupling to synthesize pharmacologically intriguing compounds.

The potential application of a new catalyst to carry out the reaction of Mizoroki-Heck in aqueous medium was discussed by Khan et al. They specifically applied the aforementioned method to functionalize pyrene derivatives. Considering that it is difficult to successfully carry out Mizoroki-Heck cross-coupling reactions in water using effective heterogeneous catalysts to produce C-C bonds, Khan and coworkers were able to catalyze, in water, Mizoroki-Heck cross-coupling reactions using a highly reduced graphene oxide (HRG) immobilized palladium nanoparticle (Pd-NPs)-based catalyst (HRG-Py-Pd). Amino pyrene was employed during the catalyst’s preparation as a functionalized ligand that provided appropriate binding sites for the efficient and uniform nucleation of Pd-NPs on the surface of HRG. As a result, the catalyst’s physical stability and water dispersibility were considerably enhanced. Accordingly, the Mizoroki-Heck cross-coupling reactions of several aryl halides with acrylic acid in a water solution were used to examine the catalytic capabilities of HRG-Py-Pd. Likewise, HRG-Py-catalytic Pd’s effectiveness was compared to that of its non-functionalized counterparts, HRG-Pd and pure Pd-NPs. The coupling reaction of 4-bromoanisol and acrylic acid in a water solution was accomplished employing the HRG-Py-Pd nanocatalyst in a relatively small time (3 h), using a limited amount of the catalyst (3 mg). In contrast, pure Pd-NPs provided lesser conversion (92%) considering the identical reaction, which necessitated a lengthy period of time to react and a significant quantity of catalyst (5.3 mg). When 3 mg of Pd-NPs were utilized that was adequate to induce a 99% conversion for HRG-Py-Pd, the conversion was further reduced to barely 40%. HRG-Pd, however, was unsuccessful and failed to produce any conversion, even when a large amount of catalyst was used, and the reaction period was prolonged. The accumulation of Pd-NPs, which decreased the catalyst’s capacity to disperse in water, is what prevents the HRG-Pd from promoting coupling processes. Because of the smart functionalization, HRG-Py-Pd has high aquatic stability, making it possible to undertake various organic reactions in water that would not have been conceivable otherwise.

Rivero Berti et al. reported the production, purification, and encapsulation of Violacein in a nanostructured lipid carrier (NLC). Considering that Violacein, a purple water-insoluble pigment produced by *Chromobacterium violaceum* and other microbes, showed an interesting pharmacological profile, also including anticancer action, the proposed approach is of extreme utility for the delivery of this water-insoluble compound. The NLC is constituted by the solid lipid myristyl myristate (a capric- and caprylic-acid-based oily lipid combination), and the P188, a surfactant poloxamer. To generate an active release system, inactive lipase from *Rhizomucor miehei* was added to NLC-Violacein. Finally, the printing Violacein encapsulation efficiency exceeded 90%. At pH = 7.4, the mesh containing NLC-lipase demonstrated a kinetic release of the biodye that was 20% faster than that without the enzyme. At pH = 5.0, when the lipase is not active, both Violacein kinetic releases showed patterns that were comparable. In addition to the biologically synergistic actions of Violacein and lipase reported, cytotoxic experiments employing the cancer cells A549 and HCT-116 demonstrated strong antitumor action of the developed complex.

Qi et al. reported a straightforward synthesis of novel androgen receptor (AR) antagonists potentially useful in prostate cancer. The use of AR-targeted therapeutics can block androgen and slow the growth of cancer cells, but they could also lead to severe resistance issues. In order to develop new AR antagonists, 22 different kinds of arylpiperazine derivatives were developed. Among these compounds, some not only exhibited strong antagonistic activity (>55% inhibition) and binding affinities (IC_50_ < 3 μM) to AR but also exhibited a significant inhibitory activity toward the LNCaP cell line than the PC-3 cell line. Interestingly, compound **21** showed the best antagonistic potency (76.2% inhibition) and binding affinity to AR (IC_50_ = 0.65 μM). The computational investigation highlighted the possible binding mode of these derivatives within the AR binding site. Overall, the findings offered experimental strategies to develop new arylpiperazine containing-compounds that are effective AR antagonists.

Regarding the review articles, two papers have been published in the Research Topic. The first work was focused on the green chemistry of chalcones, a useful source of privileged core structures for drug discovery, while the second reports the attempts to obtain levoglucosenone (LGO) through a bio-based platform with implications in drug discovery.

Marotta et al. focused their review of the literature on the sustainable usage of sources, which is crucial in many industrial sectors such as the pharmaceutical industry. However, the issue of sustainability must be taken into account during the entire process of the drug discovery trajectory, not just during the production phase. The efficiency and speed of a more sustainable drug discovery pipeline are being boosted by the ongoing advancements in the disciplines of green chemistry and the application of artificial intelligence (AI). In this context, the authors reviewed the most recent environmentally friendly and sustainable synthetic methods for creating and derivatizing chalcones, a crucial class of privileged structures and building blocks for the generation of novel molecules with interesting pharmacological profiles. In order to filter the results using the term “green chemistry,” the word “chalcone” was used as a keyword in the SciFinder database for the literature search (2017–2022), and the keywords “chalcone” and “green” were used in the Reaxys database. Particularly, in the past few years, chalcones have been employed to develop different biologically active heterocyclic scaffolds, enabling more sustainable access to a wider range of scaffolds. The most appealing procedures for derivatizing and preparing chalcone derivatives are flow chemistry and biocatalysis, which have considerable potential. Another crucial element is the use of AI to prioritize the synthesis of particular molecules. Based on the cases reported in this review and the advances in computer-aided techniques expected in the next decade, the authors anticipate that *in silico* methods will be employed to synthesize focused chemical libraries, as in the cases of chalcone-based compounds presented. This will hasten the drug discovery process, increase environmental awareness, and decrease the requirement for experimental testing.

The bio-privileged LGO compound, which can be manufactured on a massive scale from waste biomass, was studied by Camp and Greatrex. This chiral building block was employed to synthesize previously challenging building blocks including enantiopure butenolides, dihydropyrans, modified cyclopropanes, deoxy-sugars, and ribonolactones by well-known chemical methods. LGO is a great starting point for synthesizing biologically active molecules, such as those with antiinflammatory, anticancer, and antimicrobial properties. As a result, it is anticipated that the creation of reactions for the LGO ring-system and its widespread availability would open up more chances to access both established and novel bioactive chemical entities. This review is focused on the conversion of LGO to molecules with pharmacological potential, and provides upcoming research ways linked to the mentioned compound.

In conclusion, we would like to express our gratitude to all of the authors and co-authors for their significant contributions to this Research Topic in Frontiers in Chemistry, to all of the reviewers for their insightful work in assessing the presented papers, and to the editorial staff of Frontiers for the support. The success of this Research Topic was greatly reliant on all of these collective efforts, seen as a whole. We anticipate that this subject will aid green synthesis in drug development progress, and we hope that it will serve as an educational and inspirational tool for researchers and students. The following URL provides free access to the mentioned Research Topic https://www.frontiersin.org/research-topics/24085/advances-in-green-synthesis-for-drug-discovery.

